# Usefulness of TNFR1 as biomarker of intracranial aneurysm in patients with spontaneous subarachnoid hemorrhage

**DOI:** 10.2144/fsoa-2019-0090

**Published:** 2019-11-05

**Authors:** Reyes de Torres, Fernando Mancha, Alejandro Bustamante, Patricia Canhao, Isabel Fragata, Joan Montaner

**Affiliations:** 1 Neurology Unit, Hospital Virgen Macarena, Seville, Spain; 2 Neurovascular Laboratory, Instituto de Biomedicina de Sevilla, Seville, Spain; 3 Neurovascular Research Laboratory, Institut de Recerca, Hospital Universitari Vall d’Hebron, Universitat Autonoma de Barcelona, Barcelona, Spain; 4 Neuroradiology Department, Centro Hospitalar Lisboa Central, Lisboa, Portugal; 5 Neuroradiology Department, Centro Hospitalar Lisboa Norte, Lisboa, Portugal; 6 Faculdade de Medicina, Instituto de Medicina Molecular, Universidade de Lisboa, Lisboa, Portugal

**Keywords:** aneurysm, biomarker, spontaneous subarachnoid hemorrhage, stroke, TNFR1

## Abstract

**Aim:**

To determine the utility of TNF-α receptor (TNFR1) as a biomarker for the presence of aneurysms in patients with acute subarachnoid hemorrhage (SAH).

**Patient & methods:**

This is a prospective study in patients with acute spontaneous SAH. Arterial blood from catheter near aneurysm and peripheral venous blood samples are collected. TNFR1 levels were analyzed in patients with and without aneurysm.

**Results:**

80 patients were included, 58 were analyzed. 41 patients (70.7%) had an aneurysm. Venous TNFR1 levels >1658 pg/ml had 46.3% sensitivity and 94.1% specificity for aneurysms presence. TNFR1 >1658 pg/ml was also an independent predictor for its presence (odds ratio = 12.03 [1.13–128.16]; p = 0.039).

**Conclusion:**

High levels of TNFR1 in peripheral venous blood are associated with the presence of aneurysm in patients with acute SAH.

## Lay abstract

Subarachnoid hemorrhage (SAH) is a neurological emergency, in many cases caused by the rupture of a cerebral aneurysm. Usually aneurysms are detected in imaging tests, but sometimes they can be very small and go unnoticed. TNF-α is an inflammatory biomarker related to the presence and rupture of intracranial aneurysms and its receptor, TNFR1, could be detected in peripheral blood. This study demonstrates that elevated peripheral blood TNFR1 values are related to the presence of intracranial aneurysms in patients with acute subarachnoid hemorrhage. Following further research, it could become a useful tool for detecting small aneurysms in addition to conventional imaging tests.

Spontaneous subarachnoid hemorrhage (SAH) is a subtype of hemorrhagic stroke with a high number of complications leading to elevated rates of mortality and important sequelae among survivors. Up to 80% of cases are caused by the rupture of an intracranial aneurysm [1]. Therefore, to have easy tools to point to patients with an underlying aneurysm following SAH would be of interest since management differs in those cases.

The role of TNF-α as a pro-inflammatory biomarker and its relationship to the growth and rupture of intracranial aneurysms is already widely known and described in the literature [2–5].

TNF-α basically performs its function through a system of at least two ligands (soluble and transmembrane) and two receptors TNF-α receptor (TNFR1 and TNFR2), which are expressed and regulated in most cell types. TNFR1 is expressed in the walls of the intracranial aneurysms and also soluble-TNFR1 can be detected in peripheral blood [6]. TNFR1 is known to be a strong indicator of the TNF-α pathway due to its prolonged persistence in blood, and it can be used as a biomarker of TNF-α activity [7].

Its main function is related to the activation of the transcription factors NF-κB and AP-1 and the expression of genes encoding cytokines, chemokines, anti-apoptotic and cell survival molecules, amplifying several signaling pathways leading to inflammation, apoptosis and tissue degradation.

In animal models, specimens that do not express TNFR1 on the walls of aneurysms have suppressed aneurysm growth compared with those that do express the receptor [8]. Therefore, we hypothesize that due to its relationship with aneurysm formation and the possibility of detecting elevated values of this receptor in peripheral blood, TNFR1 could be used as a blood biomarker to predict the presence of ruptured aneurysms in patients with SAH and for the screening of asymptomatic intracranial aneurysms in patients with suggestive family history.

The purpose of this study is to determine cut-off values of TNFR1 in peripheral venous and in arterial blood collected near aneurysm via endovascular catheter, that might be indicative of the presence of aneurysm in patients with SAH in the acute phase.

## Methods

### Patients

All patients with acute SAH in a University Hospital between May 2013 and November 2014 were enrolled in a prospective cohort study [9]. Institutional review board approval was obtained. Inclusion criteria were: age >18 years; acute nontraumatic SAH diagnosed by computed tomography angiography (CTA) and/or lumbar puncture performed within the first 72 h of SAH; imaging studies performed within the first 72 h of SAH; informed consent obtained from patient or legal representative. We included all patients from whom both peripheral venous blood and central arterial blood samples were collected. All patients had conventional digital subtraction angiography (DSA) upon admission, confirming the presence of at least one aneurysm.

### Clinical data

Demographic data and clinical presentation were collected from the patients’ medical records. Neurological status at admission was evaluated using the Glasgow Coma Scale (GCS), World Federation of Neurosurgeons scale (WFNS) and Hunt & Hess grade (HH). The functional outcome of the patients was recorded during 6-month follow-up by using the modified Rankin Scale (mRS). The presence and location of aneurysm, and surgical/endovascular treatment were recorded. The amount of blood on admission CT was assessed using the modified Fisher scale.

### Laboratory

Blood samples were collected at the time of diagnostic DSA, at <24 h from admission, and at <72 after SAH onset. Two samples were collected simultaneously: one from peripheral venous access, and one from the catheter used in the diagnostic angiography. Arterial blood was collected from the main parent artery of the aneurysm, if any, or from a vertebral artery in perimesencephalic hemorrhages. Blood from EDTA tubes was centrifuged at 3000 × *g* (15 min), and plasma was frozen at -80°C until analysis. TNFR1 was measured by (ELISA, R&D Systems, MN, USA) according to manufacturer’s instructions. All samples were tested in duplicate, and the valid coefficient of variation was <20%. Results were expressed in pg/ml. Interassay variation was determined by testing two-times in every plate a commercial internal control (human serum type AB, male, from clotted, Sigma-Aldrich, MI, USA). Staff analyzing the samples was blind to clinical information.

### Statistical analysis

Characteristics of study patients were described using the mean (standard deviation) or median (interquartile range [IQR]) for continuous variables, and frequencies (percentages) for categorical variables. As TNFR1 levels were not normally distributed in arterial nor in venous samples, nonparametric tests were used. Intergroup comparisons of TNFR1 levels were assessed with Mann–Whitney U test. Independent predictors for the presence of aneurysm were assessed by logistic regression analysis, by the enter method, including those variables associated with the presence of aneurysms in univariate analysis. For inclusion in the logistic regression models, TNFR1 levels were dichotomized based on the cut-off with the highest accuracy, identified through receiver operating characteristics curves.

All analyses were performed using SPSS statistical package, v.22.0 (IBM Corp, NY, USA).

## Results

### Demographic data

A total of 129 patients were admitted with nontraumatic SAH during the inclusion period. 80 patients fulfilled all inclusion criteria for the main prospective cohort. Finally, only 58 patients were evaluated as they had both arterial and venous blood samples obtained under suitable conditions for analysis. The mean age was 56.7 years (±16.1), and 22 (37.9%) were male. The median HH grade was 2 (1–3), 33 patients were WFNS grade I; median GCS was 14.5 (12–15). Up to 41 (70.7%) patients presented aneurysm in arteriography. Outcome data were available for 57 patients. At 3 months, 18 patients (31.8%) had mRS >2. At 6 months, 17 patients (29.8%) had mRS >2.

### Clinical characteristics of patients with aneurysm

Aneurysm group mean age was 59.8 years (±14.6) and 29 (70.7%) were women. GCS on admission was 14 (10.5–15), 20 (48.8%) patients were WFNS grade I, median HH grade was 3 (1–3.5) and median Fisher scale was 4 (4–4). Outcome data were analyzed in 40 patients and show that only 16 patients (40%) had mRS >2 at 3 months, with similar data at 6 months [15] patients (37.5%).

### Aneurysm versus nonaneurysm

Aneurysm patients were older (59.8 vs 49.5 years; p = 0.026) and mostly women (29 vs 7; p = 0.035). Clinical and radiological severity on admission was higher in the aneurysm group, both on the HH grade (median 3 vs 1; p = 0.022) and on the Fisher scale (median 4 vs 3; p = 0.019). In the same way, the outcome was worse in aneurysm group with higher number of patients with mRS >2 at discharge (46.3 vs 12.5%; p = 0.017) and at third month (40 vs 11.8%; p = 0.036). A toward significance was found in patients with mRS >2 at month 6 (37.5 vs 11.8%; p = 0.052; Table 1).

**Table 1.** Descriptive analysis of the cohort and univariate analysis for aneurysm.

		All patients (N = 58)	Aneurysm (N = 41)	No aneurysm (N = 17)	p-value
Sex (male)		22 (37.9)	12 (29.3)	10 (58.8)	0.035
Age (years)		56.7 ± 16.1	59.8 ± 14.6	49.5 ± 17.5	0.026
GCS score		14.5 (12–15)	14 (10.5–15)	15 (15–15)	0.059
WFNS	I	33 (56.9)	20 (48.8)	13 (76.5)	0.201
	II	10 (17.2)	7 (17.1)	3 (17.6)	
	III	1 (1.7)	1 (2.4)	-	
	IV	9 (15.5)	9 (22.0)	-	
	V	5 (8.6)	4 (9.8)	1 (5.9)	
HH grade		2 (1–3)	3 (1–3.5)	1 (1–2)	0.022
Fisher grade		4 (3–4)	4 (4–4)	3 (3–4)	0.019
mRS >2 at discharge		21/57 (36.8)	19/41 (46.3)	2/16 (12.5)	0.017
mRS >2 at 3rd month		18/57 (31.6)	16/40 (40)	2/17 (11.8)	0.036
mRS >2 at 6th month		17/57 (29.8)	15/40 (37.5)	2/17 (11.8)	0.052
TNFR1 arterial (pg/ml)		1345.9 (1097.6–1946.2)	1530.6 (1144.6–2031.0)	1210.6 (1056.3–1448.3)	0.209
TNFR1 venous (pg/ml)		1437.2 (1121.1–1982.3)	1650.4 (1125.3–2216.2)	1195.1 (1078.3–1428.3)	0.074

Results are expressed as N (%) for categorical variables and as mean ± standard deviation or median (interquartile range) for continuous variables, depending on data distribution.

GCS: Glasgow Coma Scale; HH: Hunt & Hess grade; mRS: Modified Ranking scale; TNFR1: TNF-α receptor; WFNS: World Federation of Neurosurgeons scale.

### TNFR1 & aneurysm presence

**Figure 1.**
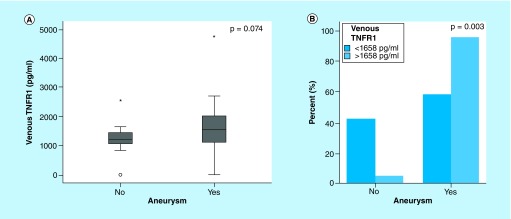
Venous TNF-α receptor levels in patients with and without aneurysms. **(A)** Boxplots represent median (interquartile range) of TNFR1 in patients with and without aneurysm. **(B)** Rates of patients with and without aneurysms among patients with TNFR1 <1658 pg/ml (light bars) and TNFR1 >1658 pg/ml (dark bars). *Outlier values. TNFR1: TNF-α receptor.

**Figure 2.**
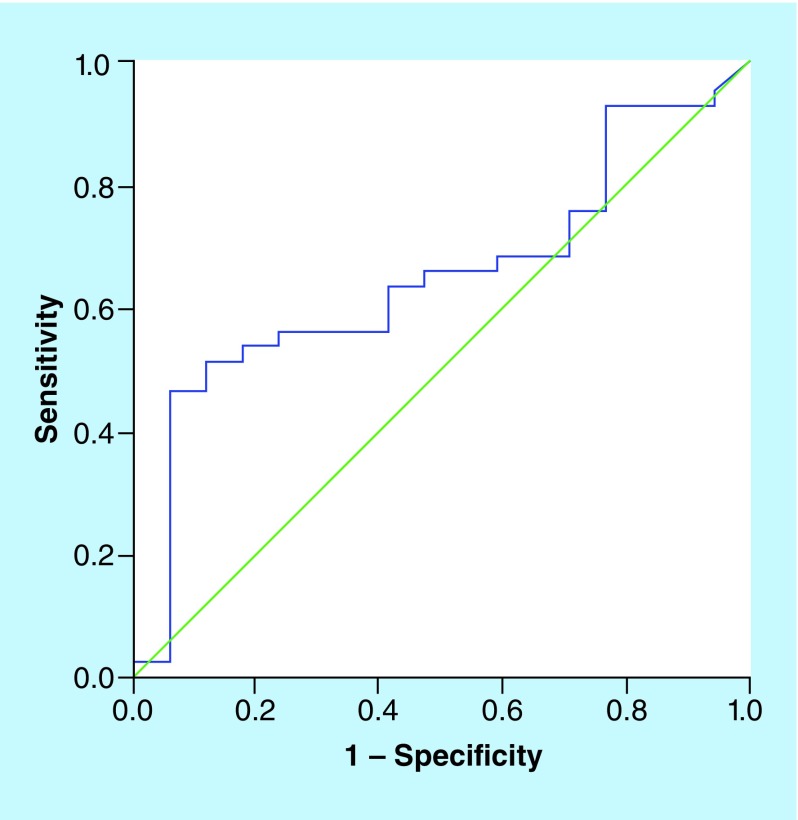
Receiver operating characteristics curve analysis and area under curve for the predictive value of venous TNF-α receptor for intracranial aneurysm presence.

Venous levels of TNFR1 were higher than arterial levels (1437 vs 1345 pg/ml); both values were strongly correlated (R = 0.706, p < 0.0001). Venous TNFR1 tended to be higher in those patients with aneurysms (1650 vs 1195 pg/ml; p = 0.074). Cut-off point of venous TNFR1 >1658 pg/ml had 46.3% sensitivity and 94.1% specificity for the presence of aneurysms. In fact, those patients with TNFR1 >1658 pg/ml had higher rates of aneurysm than patients with lower levels (Figure 1). In logistic regression analysis, after adjustment by age, sex, GCS, HH grade and Fisher scale, TNFR1 >1658 pg/ml was the only independent predictor for the presence of aneurysm (odds ratio = 12.03 [1.13–128.16]; p = 0.039) (Table 2). The area under curve significance for venous TNFR1 values detecting intracranial aneurysm presence is 0.074 (95% CI: 0.502–0.798) (Figure 2).

**Table 2.** Logistic regression analysis for aneurysm.

Variable	Odds ratio	95% CI	p-value
Sex (male)	0.21	0.03–1.27	0.089
Age	1.00	0.93–1.08	0.937
GCS	1.59	0.79–3.19	0.190
HH	4.34	0.87–21.6	0.073
Fisher grade	2.78	0.80–9.67	0.108
TNFR1 > 1658.7 pg/ml	12.03	1.13–128.16	0.039

GCS: Glasgow Coma Scale; HH: Hunt & Hess grade; TNFR1: TNF-α receptor.

## Discussion

This study demonstrates the potential role of TNFR1 as a biomarker to identify aneurysm presence in cases of SAH. Although many cases are caused by the rupture of an intracranial aneurysm, sometimes detection using imaging tests such as CT or MR angiography is difficult, even after rupture, because they can be quite small.

The possibility of detecting TNFR1 levels in peripheral blood as a biomarker for the presence of aneurysms in patients with SAH could make the diagnosis of cerebral aneurysms easier and faster, due to the high specificity it presents. In some cases, its use could even make it possible to avoid complementary diagnostic tests and directly access endovascular treatment. The presence of higher levels in peripheral venous blood compared with arterial blood would add simplicity to the determination of this biomarker in real-life conditions. It is possible that all SAH patients have higher TNFR1 level and that ones with aneurysm even higher. If we check some controls group from the literature, using similar ELISAs, they have lower TNFR1 level in a control population for psychiatric disorders (901 pg/ml) and in a control population for Alzheimer disease (1292 pg/ml), supporting the fact that all SAH patients have higher TNFR1 level than controls and the ones with aneurysm even higher [10,11].

Beyond SAH, intracranial aneurysms may be asymptomatic and affect 5–10% of the general population. They usually do not produce clinical symptoms and are usually detected after rupture [12]. Hypertension, smoking and alcohol abuse are modifiable risk factors involved in the formation and rupture of aneurysms, related pro-inflammatory and immunological factors have also been described [13]. However, the presence of predisposing family history, especially in first-degree relatives, is the strongest nonmodifiable risk factor [14]. In these patients, the detection of TNFR1 levels could help to diagnose the presence of aneurysms as screening method in direct relatives. It is possible the test will be useful when CTA is negative, obviating the need for additional DSA, or in small ones or when doubts appear, where it could act as a supportive test. If positive, radiologists would be advised that an aneurysm may be present and that perhaps some therapeutic procedure will be needed following confirmatory diagnosis.

In the last few years, various monoclonal antibody therapies have been developed with anti-TNF-α targets such as infliximab, adalimumab or etanercept, used in the treatment of rheumatoid arthritis, Crohn’s disease, ankylosing spondylitis, psoriasis and other inflammatory diseases, despite the risk of related side effects [15]. The study and development of specific therapies against TNFR1 could be a therapeutic strategy to control growth and avoid the rupture of asymptomatic intracranial aneurysms.

## Limitations

To bring these results to the clinics, many confirmatory and validation studies are needed with additional groups, such as healthy matched controls and patients with suspicion of aneurysm that did not suffer SAH in which a high sensitive test might be used to screen the presence of aneurysm among those at higher risk with vascular risk factor or familiar history of aneurysm. To explore the value of TNFR1 in those groups both at the venous and arterial level might be of interest to better understand its role in aneurysm formation dynamics. Although the presence of TNFR1 in the aneurysm has been elegantly demonstrated [8], the relationship between local production and the circulating levels has not been studied, to our knowledge, and this would have to be investigated in experimental studies. If confirmed, this might be tested in humans with resected aneurysms.

All of the above should be investigated before any anti-TNF therapies are tested in humans to modify brain aneurysm natural history.

## Conclusion

Although these results are promising, further studies should be performed to replicate our findings in large SAH populations and to determine TNFR1 values in patients with asymptomatic intracranial aneurysms and its behavior in the case of aneurysm growth, in order to establish patterns and treatments that could avoid their rupture.

## Future perspective

Blood biomarkers are becoming a very useful diagnostic tool as they help us to select the most appropriate diagnostic tests for each patient, avoiding unnecessary procedures. The use of TNFR1 as a biomarker for the presence of aneurysms in patients with SAH might help to complement the diagnostic approach for these patients, especially in those with small aneurysms in which an Angio-RMN or cerebral arteriography may appear normal. It is probable that such a technique will not replace the use of imaging techniques as diagnostic gold standard, but it could support the diagnosis in doubtful cases. If this blood biomarker is combined with others in a panel that improves sensitivity close to 100%, it might become ideal for use as a screening tool to rule out the presence of aneurysm in high-risk populations (those with vascular risk factors, familiar cases, etc/). Moreover, among people with known aneurysms, it would be interesting to know if TNFR1 levels are related to the growth or rupture of such aneurysms.

Due to its expression on the walls of the aneurysm and the direct relationship between increased TNF-α levels and the growth and rupture of intracranial aneurysms, this biomarker could be used as a therapeutic target. Anti-TNF-α biologic therapies may be especially effective in asymptomatic patients with unruptured intracranial aneurysms.

Along the same lines, it would be necessary to study and titrate TNFR1 values in patients with the presence of asymptomatic intracranial aneurysms confirmed through imaging to determine, after a possible biological treatment, if the growth of the aneurysm stops or even decreases, as well as to understand how the biomarker value changes after that treatment.

Perhaps in the near future anti-TNF-α biologic therapies might provide a safe alternative to intracranial surgery or endovascular approaches, with a lower complication rate, especially in young patients with familial intracranial aneurysms.

**Summary points**

- TNF-α is a pro-inflammatory biomarker related to aneurysm growth and rupture.
- TNF-α receptor (TNFR1) is located in intracranial aneurysm walls and can be detected in arterial and venous peripheral blood.
- Due to its relationship with aneurysm formation and the possibility of detecting elevated values of this receptor in peripheral blood, TNFR1 could be used as a blood biomarker to predict the presence of intracranial aneurysms.
- In our sample, patients with acute subarachnoid hemorrhage and aneurysm presence are older, mostly women, with higher clinical-radiological severity and worse outcome, compared with nonaneurysm patients.
- TNFR1 has higher levels in patients with aneurysms present, with a cut-off point of venous TNFR1 >1658 pg/ml with 46.3% sensitivity and 94.1% specificity.
- Venous levels of TNFR1 were higher than arterial, making easy to use its determination as a screening method for the presence of aneurysms in patients with acute subarachnoid hemorrhage.
- Its determination could be useful as a screening tool for the detection of intracranial aneurysms in asymptomatic patients with suggestive familiar history.
- Developing specific therapies against TNFR1 could be a therapeutic strategy to control growth and avoid the rupture of asymptomatic intracranial aneurysms.
